# Hybrid Models Based on Fusion Features of a CNN and Handcrafted Features for Accurate Histopathological Image Analysis for Diagnosing Malignant Lymphomas

**DOI:** 10.3390/diagnostics13132258

**Published:** 2023-07-04

**Authors:** Mohammed Hamdi, Ebrahim Mohammed Senan, Mukti E. Jadhav, Fekry Olayah, Bakri Awaji, Khaled M. Alalayah

**Affiliations:** 1 Department of Computer Science, Faculty of Computer Science and Information System, Najran University, Najran 66462, Saudi Arabia; balawaji@nu.edu.sa; 2 Department of Artificial Intelligence, Faculty of Computer Science and Information Technology, Alrazi University, Sana’a, Yemen; 3 Shri Shivaji Science & Arts College, Chikhli Dist., Buldana 443201, India; 4 Department of Information System, Faculty Computer Science and Information System, Najran University, Najran 66462, Saudi Arabia; dr.fekry_olayah@yahoo.com; 5 Department of Computer Science, Faculty of Science and Arts, Sharurah, Najran University, Najran 66462, Saudi Arabia; kmalalayah@nu.edu.sa

**Keywords:** deep learning, XGBoost, fusion features, DT, malignant lymphoma, ACO, GVF

## Abstract

Malignant lymphoma is one of the most severe types of disease that leads to death as a result of exposure of lymphocytes to malignant tumors. The transformation of cells from indolent B-cell lymphoma to B-cell lymphoma (DBCL) is life-threatening. Biopsies taken from the patient are the gold standard for lymphoma analysis. Glass slides under a microscope are converted into whole slide images (WSI) to be analyzed by AI techniques through biomedical image processing. Because of the multiplicity of types of malignant lymphomas, manual diagnosis by pathologists is difficult, tedious, and subject to disagreement among physicians. The importance of artificial intelligence (AI) in the early diagnosis of malignant lymphoma is significant and has revolutionized the field of oncology. The use of AI in the early diagnosis of malignant lymphoma offers numerous benefits, including improved accuracy, faster diagnosis, and risk stratification. This study developed several strategies based on hybrid systems to analyze histopathological images of malignant lymphomas. For all proposed models, the images and extraction of malignant lymphocytes were optimized by the gradient vector flow (GVF) algorithm. The first strategy for diagnosing malignant lymphoma images relied on a hybrid system between three types of deep learning (DL) networks, XGBoost algorithms, and decision tree (DT) algorithms based on the GVF algorithm. The second strategy for diagnosing malignant lymphoma images was based on fusing the features of the MobileNet-VGG16, VGG16-AlexNet, and MobileNet-AlexNet models and classifying them by XGBoost and DT algorithms based on the ant colony optimization (ACO) algorithm. The color, shape, and texture features, which are called handcrafted features, were extracted by four traditional feature extraction algorithms. Because of the similarity in the biological characteristics of early-stage malignant lymphomas, the features of the fused MobileNet-VGG16, VGG16-AlexNet, and MobileNet-AlexNet models were combined with the handcrafted features and classified by the XGBoost and DT algorithms based on the ACO algorithm. We concluded that the performance of the two networks XGBoost and DT, with fused features between DL networks and handcrafted, achieved the best performance. The XGBoost network based on the fused features of MobileNet-VGG16 and handcrafted features resulted in an AUC of 99.43%, accuracy of 99.8%, precision of 99.77%, sensitivity of 99.7%, and specificity of 99.8%. This highlights the significant role of AI in the early diagnosis of malignant lymphoma, offering improved accuracy, expedited diagnosis, and enhanced risk stratification. This study highlights leveraging AI techniques and biomedical image processing; the analysis of whole slide images (WSI) converted from biopsies allows for improved accuracy, faster diagnosis, and risk stratification. The developed strategies based on hybrid systems, combining deep learning networks, XGBoost and decision tree algorithms, demonstrated promising results in diagnosing malignant lymphoma images. Furthermore, the fusion of handcrafted features with features extracted from DL networks enhanced the performance of the classification models.

## 1. Introduction

Cancer is one of the deadliest diseases due to abnormal cell division and abnormal reproduction. Malignant lymphoma is considered a disease that leads to death due to the exposure of lymphocytes to malignant tumors, which represent 50% of the most severe types of blood diseases [1]. The transformation of cells from indolent B-cell lymphoma to diffuse B-cell lymphoma (DBCL) is life-threatening. There are many types of lymphoma that range from slow to aggressive and rapidly spreading [2]. Most types of lymphoma arise from two types of white blood cells: B and T lymphocytes. B-cell lymphoma is one of the most malignant types of lymphoma [3]. Common types of malignant B lymphomas are follicular lymphoma (FL), chronic lymphocytic leukemia (CLL), and mantle cell lymphoma (MCL). There are clinical symptoms for each type of malignant lymphoma. The FL lymphocyte accumulation causes lumps in the neck, thighs, and armpits [4]. CLL is considered one of the most severe types of lymphoma leukemia due to disorders in the ability of the bone marrow to produce white blood cells. CLL develops slowly, and symptoms may be delayed and not appear until years later. CLL appears in some body parts, such as the spleen, liver, and lymph nodes. CLL cells transform into DBCL, which leads to rapid deterioration and median patient survival time of 12 months [5]. The MCL type is a non-Hodgkin lymphoma that grows in the mantle, causing abnormal B cell growth and abnormal lymph node masses. This type spreads to the liver, bone marrow, and digestive system. Patients with lymphomas undergo lymph node or pathological biopsies to confirm the diagnosis [6]. Because of the multiplicity of types of malignant lymphomas, manual diagnosis by pathologists is difficult, and the analysis of physicians differs. Biopsies taken from the patient are the gold standard for analysis. The biopsy is placed on a slide stained with hematoxylin and eosin (H&E) for analysis. Immunohistochemical (IHC) is used to diagnose the type of malignant lymphoid tissue by examining the characteristics of the biopsy [7]. However, manual diagnosis still has many limitations. First, observing pathological tissues is hard and tedious work that requires time and effort. Secondly, the gap between patients and skilled doctors is still an obstacle, especially in developing countries. Third, because of the diversity of malignant lymphomas, the difference in diagnosis between doctors and the inconsistency between their diagnoses can lead to the patient’s death. Hence, there is an urgent need for automated diagnostics using AI techniques that address the limitations of manual diagnosis [8]. Glass slides under a microscope are converted into WSI to be analyzed by AI techniques through biomedical image processing [9]. Deep learning networks have played a significant role in recent years in analyzing WSI images of pathological tissues of lymph nodes. DL networks feature many convolutional layers to analyze WSI images through many filters whose size varies from one convolutional layer to another [10]. After analyzing the WSI images by convolutional layers, the features extracted are high-dimensional, and the model contains millions of connections and neurons, which requires complex calculations. DL networks provide pooling layers to reduce connections and neurons. In this study, several hybrid systems with various instruments were designed to analyze histopathological WSI images for diagnosing malignant lymphomas and distinguishing between their types. The ACA segmentation algorithm was applied to extract the affected pathological tissue cells, isolate them from the rest of the image, and save them as inputs to the CNN models. Despite the diversity of types of malignant lymphoma, the clinical characteristics in the early stages are similar, which constitutes an obstacle for doctors. Thus, this study extracted features from several DL networks and combined them to form more efficient feature vectors. The color, geometric, and texture features were extracted by many methods and incorporated into feature vectors called handcrafted features. Finally, we obtained more accurate and efficient features to distinguish between types of malignant lymphomas by combining the features of the fused DL network with the features of the handcrafted. Thus, many fused feature vectors were obtained and classified by XGBoost and the DT networks.

The rationale for utilizing pre-trained deep learning models in this study stems from the need for automated diagnostics that can overcome the limitations of manual diagnosis. By leveraging the capabilities of deep learning networks to extract high-dimensional features from WSI images and combining them with handcrafted features, the proposed hybrid system aims to provide more accurate and efficient diagnoses, ultimately improving patient outcomes in the challenging domain of malignant lymphomas.

The novelty of this article lies in the development of a hybrid system that combines deep learning networks with handcrafted features for the analysis of histopathological WSIs in the diagnosis of malignant lymphomas and distinguishing between their types. While deep learning networks have been employed in analyzing WSI images of pathological tissues in recent years, this study extends the approach by integrating additional handcrafted features such as color, geometric, and texture features. By fusing the features extracted from deep learning networks with the handcrafted features, the proposed system aims to provide more accurate and efficient feature vectors for improved differentiation between various types of malignant lymphomas. Furthermore, the classification of the fused feature vectors using XGBoost and DT networks adds an additional layer of sophistication to the analysis process. This novel hybrid approach has the potential to enhance automated diagnostics and address the limitations associated with manual diagnosis, ultimately contributing to improved patient outcomes in the field of malignant lymphomas.

In the context of early diagnosis of malignant lymphomas, the combination of features from two CNN models and handcrafted features from WSI images provides a comprehensive and robust representation of the underlying pathology. CNN models are deep learning architectures specifically designed for image analysis tasks and have shown remarkable success in various medical imaging applications. They can automatically learn relevant features from input images and capture intricate patterns and structures. Additionally, handcrafted features extracted from WSI images are engineered by domain experts to capture specific characteristics of lymphoma pathology. These features can include morphological, textural, and statistical attributes that are manually defined based on prior knowledge and expertise. By combining the strengths of XGBoost and DT algorithms with the feature representations from CNN models and handcrafted features from WSI images, the developed system can effectively detect early signs of malignant lymphomas. The ensemble approach of XGBoost allows for better prediction performance by combining multiple models, while the decision tree algorithms provide interpretability and flexibility in decision making. Overall, the main contribution of this algorithmic combination is an advanced diagnostic system that leverages the power of machine learning and deep learning techniques to enhance the accuracy and efficiency of early diagnosis for malignant lymphomas, thereby facilitating timely treatment and improved patient outcomes.

The primary contributions of this study are as follows:

- Passing histological images of malignant lymphomas through two overlapping Gaussian and Laplacian filters.
- Applying a hybrid model between DL networks with XGBoost and DT networks based on the GVF algorithm to diagnose malignant lymphoma images effectively.
- Diagnosis of malignant lymphoma images by XGBoost and DT networks with fused features of MobileNet-VGG16, VGG16-AlexNet, and MobileNet-AlexNet based on the ACO algorithm for features selection.
- Efficient and accurate diagnosis of malignant lymphoma images by XGBoost and DT grids with fused features of MobileNet-VGG16, VGG16-AlexNet, and MobileNet-AlexNet and handcrafted features based on the ACO algorithm for features selection.

The remaining sections of this paper are structured in the following manner:

In Section 2, various techniques and results from previous studies regarding the diagnosis of malignant lymphomas are discussed. Section 3 elaborates on the methods and materials employed in this study to analyze images of malignant lymphomas. The outcomes of the proposed systems are summarized in Section 4. Section 5 evaluates and compares the performance of the proposed systems with the results presented in Section 2. Finally, the study is concluded in Section 5.

## 2. Related Work

Here, we discuss techniques and tools applied in previous studies related to analyzing images of malignant lymphomas to distinguish between their types.

Irshaid et al. [11] trained CNNs on WSI smear images to predict the progression and spread of lymphoma. Morphological parameters were recorded along with features being extracted to analyze the transformation of B-cell lymphoma cells into diffuse large B-cell lymphoma. The results of all the CNNs improved on the characteristics extracted by the doctors, reaching an AUC of 92.3%. Xia et al. [12] constructed a CNN by fluid-attenuation, weighted axial rotation inversion, and an apparent diffusion map by collecting predictions of single CNNs to diagnose lymphoma images. The CNN with CE-T1WI achieved an accuracy of 88.4%, a sensitivity of 93.4%, and an AUC of 95.6%. Savas et al. [13] used a CNN-LSTM method for the analysis of WSI images of lymphomas. The dataset images were augmented by the data augmentation method and analyzed through a mixed network between the CNN and LSTM with RGB and grayscale images. CNN-LSTM achieved an accuracy of 96.65%, a sensitivity of 96.51%, and an F1 score of 96.67%. Miyoshi et al. [14] used deep learning networks for image classification of histological WSI images of malignant lymphomas. Hematologists annotated the WSI images, and image regions were sectioned with several magnification factors. The network achieved an accuracy of 94% with images with a 5× magnification, while it achieved an accuracy of 92% with images with a 40× magnification. Li et al. [15] used GOTDP-MP-CNNs networks for pathologic classification of malignant B-cell lymphomas. The images were enhanced, data augmented, and features extracted and classified through three models with different layers. Syrykh et al. [16] developed Bayesian neural networks (BNNs) augmented with certainty estimation to analyze WSI images to diagnose malignant lymphomas. The BNNs had the feature of detecting small lymphoma characteristics or heterogeneous characteristics as predictors and prognosticators of malignant pictures of lymph nodes. In Zhang et al. [17], WSI images of malignant lymph nodes were analyzed by a backpropagation (BP) network with optimization by a genetic algorithm (GA). The images were improved through color uniformity, permutation, and feature extraction. The BP network achieved an accuracy of 96%, while the GA-BP achieved an accuracy of 97.7%. Reena et al. [18] used feature-based image retrieval method CNN models and traditional feature reduction learning methods for primary diagnosis of malignant lymphoma. ResNet-101 was used for feature extraction through activation feature layers. The dataset was balanced and then characterized by feature reduction to select the features for image retrieval. Euclidean distance was applied to measure the similarity of retrieved images. Zahra et al. [19] used methods for discovering the best deep learning methods for diagnosing histological images with magnification factors of 100× and 400×. The MobileNet model was the best for diagnosing histological images with a magnification factor of 100×, reaching an accuracy of 53%, while with 400× images, the Inception-V3 achieved the best resolution, at 80%. Zhang et al. [20] used a CNN model to obtain high accuracy by training the dataset based on lymph node regions through convolutional, dropout, max pooling, and dense layers. The network achieved an accuracy of 93.17% compared to 84.49% for the AlexNet model. Swiderska [21] et al. used a CNN model for automating transfusions of B lymph nodes based on HE-stained glass samples. The method works on the whole slide based on HE staining. Hence, the genetic changes are visible as morphology changes in the HE-stained sample. The method reached an accuracy of 77% and an AUC of 83%. Sheng et al. [22] used an R-CNN network to identify lymphocytes from a malignant lymphoma dataset. The dataset was used to fine-tune the pre-trained network, and then the same dataset was trained on three different networks. The network reached a rate of 96% for detecting malignant lymphocytes. Mohlman et al. [23] used a CNN to distinguish between Burkitt lymphoma and DBCL. The network performance differed based on the number of images, pixels, pixel increase, color absence, smearing, and network parameters. Gaidano et al. [24] developed machine learning networks to diagnose WSI images for DBCL collected from several sources. The images were optimized and features extracted and then classified, achieving the best network accuracy of 92.68% and sensitivity of 88.54%. Francisco et al. [25] developed a framework for extracting lymphoma biomarkers to determine their value compared to the rest of the nucleus. Artifacts were removed, and the images were passed to a U-Net model for segmentation.

Eline et al. [26] conducted a study to develop a diagnostic scoring tool for children with cervical lymphadenopathy, focusing on predicting high-grade lymphomas. They used a multivariate machine learning model that integrated the best univariate predicting factors and assigned weighting factors to each variable using logistic regression. The study resulted in a 12-factor diagnostic model that exhibited a high sensitivity of 95% and a specificity of 88% for detecting classical Hodgkin lymphoma (cHL) and non-Hodgkin lymphoma (NHL). Yuhan et al. [27] applied DL-based CAD models to classify lymph nodes and found that the DL-CNN model with the ResNet50 architecture on PET/CT images and the DL-SVM model using the ResNet50 extractor were particularly effective in diagnosing lymphomatous and metastatic lymph nodes. The inclusion of handcrafted features alongside DL-based features further enhanced the diagnostic performance. The DL-SVM model on the ResNet50 extractor showed great performance for the testing cohort, with an AUC of 0.901, accuracy of 86.96%, sensitivity of 76.09%, and specificity of 94.20%. Noriaki et al. [28] introduced a novel approach for retrieving similar cases in histopathological images of malignant lymphoma, incorporating attention-based multiple-instance learning and contrastive distance metric learning with IHC staining patterns. The method demonstrated improved performance compared to existing approaches and was validated by expert evaluations.

Through discussing the techniques of previous studies, it is noted that there is a gap in distinguishing between types of malignant lymphomas with high accuracy. Thus, this study focused on previous studies’ gaps by applying hybrid techniques based on fused features. Because of the similarity of the morphological characteristics between the different types of lymph nodes, this study focused on extracting the features fused between deep learning models and merging them with the characteristics of color, texture, and shape (radiomics) extracted using traditional methods.

Table 1 discusses the strengths and weaknesses of each previous study.

As can be seen from the table, the proposed method has several strengths over previous approaches. It uses a hybrid technique that combines features extracted from deep learning models with features extracted from traditional methods. This allows the proposed method to take advantage of the strengths of both deep learning and traditional methods. This makes the proposed method more accessible and scalable than previous approaches. The proposed method stands out by addressing the gap in accurately distinguishing between different types of malignant lymphomas. It achieves this by fusing features from deep learning models with characteristics of color, texture, and shape (radiomics) extracted from traditional methods. The inclusion of radiomics features can provide additional information for improved classification accuracy.

## 3. Materials and Methods

### 3.1. Description of the Malignant Lymphoma Dataset

**Figure 1:**
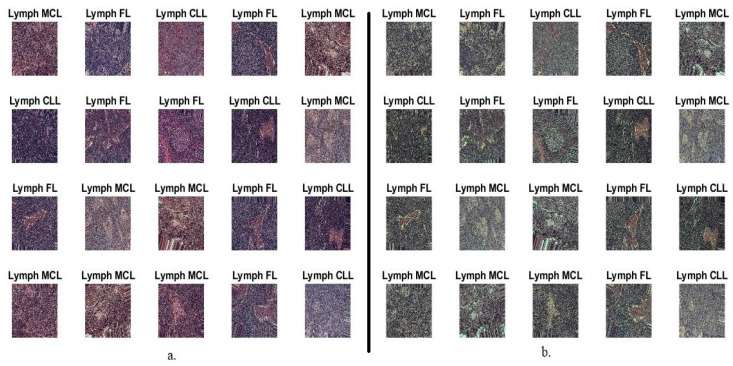
Random samples from all classes of the malignant lymphoma dataset: (**a**). original images, (**b**). improved images.

This study evaluated systems through biopsies mixed with H&E solutions on liquid-based cytology (LBC) glass slides that were converted under a microscope into WSI images. The dataset contains 15,000 WSI images divided equally among three types of malignant lymphomas, FL, CLL, and MCL [29]. The dataset is available on Kaggle for researchers and those interested, in JPEG format and image resolution 512 × 512. Figure 1a presents some dataset samples for three randomly selected classes. The dataset was divided during the system phases into 80% for training and validation and 20% of the WSI images dataset was used for system testing (80:20). Because of the balance of the dataset, where each class has 5000 images, the systems’ randomly divided each class equally into 3200 images for the training, 800 for the validation, and 1000 for the systems testing.

### 3.2. Improvement of Histopathological WSI Images

The images taken from the biopsy include some artifacts due to their mixing with blood and the different colors of the solutions, which negatively affect the next stages. So, the primary purpose of preprocessing is to remove all artifacts. We used a Gaussian filter, a preprocessing technique, to improve the histopathological WSI images, ensuring efficiency in the next stage. The presence of some artifacts in the next stage of pretreatment may clog an essential fraction of malignant lymphoma cells, making it difficult to extract features from this segment [30]. In this study, the histopathological images of WSI were optimized using Gaussian and Laplacian filters, which improve image quality and remove unwanted pixels. The study used a Gaussian filter with an operator size of 4 × 4 to remove all unwanted pixels from the WSI images and calculate the values of pixels adjacent to the target pixel using a 2D convolution operator. The filter determines the degree of smoothing by the standard deviation. Equation (1) describes the mechanism of action of the Gaussian filter to remove unwanted artifacts.

(1) $$h\left(x,y\right)=\frac{1}{2\pi \sigma^{2}}℮\frac{x^{2}-y^{2}}{2\sigma^{2}}$$

where *x* is the distance from the horizontal axis of the origin, *y* is the distance from the vertical axis of the origin, and *σ* is the standard deviation of the Gaussian distribution.

There is a low contrast in the edges of some lymphoma cells; therefore, a Laplacian filter was applied to show the edges of malignant lymphoma cells. In the Laplacian filter, the second derivative highlights the regions of change and shows the edges of the lymphoma cells, as in Equation (2) [31].

(2) $$\nabla^{2}f=\frac{\partial^{2}f}{\partial^{}x^{2}}+\frac{\partial^{2}f}{\partial^{}y^{2}}$$

where *x* and *y* are as shown in Equations (3) and (4).

(3) $$\frac{\partial^{2}f}{\partial^{}x^{2}}=f\left(x+1,y\right)+f\left(x-1,y\right)-2f\left(x,y\right)$$

(4) $$\frac{\partial^{2}f}{\partial^{}y^{2}}=f\left(x,y+1\right)+f\left(x,y-1\right)-2f\left(x,y\right)$$

Finally, the two output images of the Gaussian and Laplacian filters were merged to obtain an improved image with an improvement in the contrast of the edges of lymphoma cells, as in Equation (5).

(5) $$z(x,y)=O(x,y)-\nabla^{2}f$$

where z(x,y) is the improved WSI histopathological image.

Figure 1b shows improved images randomly selected from the dataset for three classes.

### 3.3. Gradient Vector Flow Algorithm

The segmentation stage is one of the most important and challenging stages of image processing, which must be accurate. The following stages depend on the accuracy of extracting malignant lymphocytes and isolating them from the rest of the images. Biopsy images contain malignant lymph node cells and intercellular regions. Therefore, extracting features from the whole image leads to obtaining the features of the whole image [32]. Therefore, it is necessary to separate the cells of the ROI, isolate them from the rest of the image, and send them to the deep learning and traditional methods, namely, FCH, DWT, LBP, and GLCM, for feature extraction. This study used gradient vector flow (GVF) to segment malignant lymphoma cells from WSI histological images.

The GVF method is an extended method for active contour methods. The GVF is selected by edge detection *f*(*x*, *y*) images from histopathological images *I*(*x*, *y*), as in Equation (6).

(6) f(x,y)=−Gσ(x,y)∗I(x,y)fxy

where *Gσ*(*x*, *y*) denotes a two-dimensional Gaussian function with standard deviation *σ* and statistical parameters.

GVF reduces the energy function, which depends on the smoothing, and the information depends on the parameter *μ*. The parameter *μ* depends on the noise in the image. The parameter *μ* is increased if the noise level is high. Thus, the noise around the edges will be reduced, the contour will be weakened, and the edges of the region of interest (ROI) will be shown. Equation (7) shows the arithmetic expression for the energy function.

(7) $$\varepsilon =\iint \mu (ux^{2}+vx^{2}+vy^{2})+|\nabla f{|}^{2}|g-\nabla f{|}^{2}dxdy$$

where *g* means a gradient vector flow and ∇ means a gradient operator.

**Figure 2:**
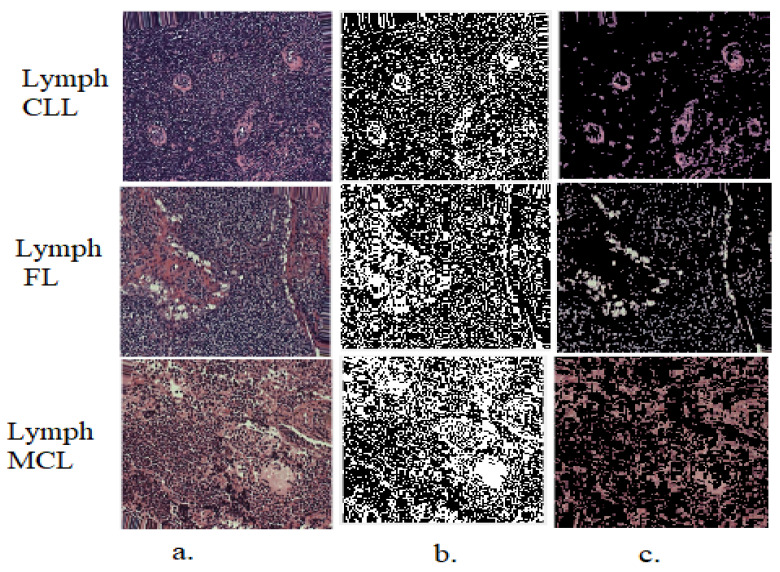
WSI images from all classes of the malignant lymphoma dataset after application of the GVF algorithm: (**a**). original images; (**b**). after segmentation; (**c**). malignant lymphoma cells (ROI).

Malignant lymphoma cell regions are segmented by specific parameters using the GVF method. This method selects the edges of malignant lymphoma cells and separates them from the rest of the image. Thus, regions of interest (malignant lymphoma cells) are obtained, isolated from the rest of the image, and sent to deep learning models and traditional methods for analysis and feature extraction. Figure 2 shows random samples of the malignant lymphoma dataset after the application of the GVF method.

### 3.4. Extract Deep Feature Maps

Deep learning models contain dozens of layers divided between convolutional layers, followed by auxiliary and pooling layers, and ending with fully connected layers. Deep learning models are distinguished by their superior ability to analyze medical images and extract hidden features that doctors do not see. The input layers receive images of size *m x n x z*, where m is the image’s width, n is its height, and z is the number of color channels. The images are resized according to each deep learning model. They are then sent to the first convolutional layer to perform processing according to the function of each convolutional layer [33]. Each deep learning model has a different number of convolutional layers. Each convolutional layer has a filter size of *f x f x z* such that the filter channels must match the channels of the input image. The convolutional layers control three parameters [34]: First, the size of the filter *f*(*t*) that wraps around the image *x*(*t*), so that all image regions are treated as in Equation (8). Second, the zero padding preserves the size of the image when the image is wrapped around a different-sized filter [35]. Third, the p-step is the number of steps the filter jumps on the image. Each convolution layer has a specific task, so it produces feature maps based on the filter; for example, one layer produces color features, another for edge features, another for geometry features, and so on. The output of each convolutional layer is an input to another layer [36].

(8) $$y\left(t\right)=\left(x*f\right)\left(t\right)=\int x\left(a\right)f\left(t-a\right)da$$

where *f*(*t*) is the filter, *x*(*t*) is the image input, and *y*(*t*) is output.

After some convolutional layers, ReLU layers handle negative values, convert them to zero, and pass positive values. Convolutional layers produce high-dimensional feature maps, which require complex computations [37]. Deep learning models provide pooling layers that reduce the dimensions of feature maps [38]. The pooling layers work in two ways. First is max pooling, which selects a group of image pixels, searches for the maximum value among the selected values, and replaces it with the group of pixels, as in Equation (9). Second is average pooling, which selects a group of image pixels, calculates its average, and replaces it, as in Equation (10) [39].

(9) $$z\left(i;j\right)={max}_{m,n=1\ldots .k}f\left[\left(i-1\right)p+m;\left(j-1\right)p+n\right]$$

(10) $$z\left(i;j\right)=\frac{1}{k^{2}}\sum_{m,n=1\ldots .k}f\left[\left(i-1\right)p+m;\left(j-1\right)p+n\right]$$

where *f* denotes the filter in the area image, *k* denotes the number of pixels, *m*, *n* denotes the matrix position, and *p* denotes the p-step.

Feature maps must represent input images and features converted to high-level convolutional layers into flat features by fully connected layers. Finally, the softmax activation function labels each image to its appropriate class. In this study, the fully connected layers were deleted from the deep learning models, and the task of the deep learning models was limited to feature extraction only [40]. The last convolutional layers of the deep learning models produced feature maps of size (7, 7, 1024), (7, 7, 512), and (13, 13, 256), for the MobileNet, VGG16, and AlexNet models, respectively. Thus, the high-level convolutional layer features were converted into flat features by the global average pooling layer and saved with sizes of 1024, 4096, and 4096 for the MobileNet, VGG16, and AlexNet models, respectively. Therefore, the sizes of the datasets after converting to features became 15,000 × 1024, 15,000 × 4096, and 15,000 × 4096 for the MobileNet, VGG16, and AlexNet models, respectively. This feature matrix was then sent to the XGBoost and DT algorithms to classify.

To extract features from WSI of malignant lymphocytes using the MobileNet, VGG16, and AlexNet models, several steps are typically taken to control the fit of these models. There follows an explanation of the general process:

Preprocessing: WSI images often require preprocessing to enhance certain characteristics or reduce noise. Preprocessing steps may include color normalization, resizing, and filtering to improve the quality and consistency of the images.

Feature Extraction: MobileNet, VGG16, and AlexNet are models that are widely used for image classification tasks. These models are trained on a malignant lymphoma dataset and have learned to extract meaningful features from images. To extract features from WSI images, the models are typically used as feature extractors. The input WSI images are passed through the MobileNet, VGG16, and AlexNet models, and the activations from one or more layers are extracted as feature representations.

Layer Selection: The selected layers from the MobileNet, VGG16, and AlexNet models play a crucial role in controlling the fit for feature extraction. Typically, the earlier layers of CNN models capture low-level features such as edges, textures, and basic shapes, while the deeper layers capture more complex and abstract features. The choice of layers depends on the specific task and the level of granularity required in the feature representation. For example, for WSI images of malignant lymphocytes, it may be beneficial to use both early and deep layers to capture both local and global information.

Dimensionality Reduction: The extracted features from the selected layers of the CNN models often have high dimensionality, which is computationally expensive and may lead to overfitting. To control the fit and reduce dimensionality, the ACO algorithm was used, which has been applied to transform the features into a lower-dimensional space while preserving their discriminative power.

### 3.5. Ant Colony Optimization Algorithm

The ACO algorithm receives the fused feature maps of the MobileNet, VGG16, and AlexNet models, selects the best features, and deletes the duplicates.

The ACO algorithm can be used to select the most important features in a dataset. The basic idea is to represent feature subsets as paths in a graph, and use the ACO algorithm to find the subset of features that maximizes the performance of a given classification or regression model [41].

The ACO algorithm can be adapted for feature selection as follows:

Initialization: Initialize the pheromone trail for each feature with a small value.

Ant movement: Release a number of artificial ants on the graph. Each ant selects features probabilistically, based on the strength of the pheromone trail on the edges corresponding to those features. The probability of selecting a feature is proportional to the pheromone trail on the corresponding edge.

Feature subset evaluation: Use the selected subset of features to train a classification or regression model and evaluate its performance on a validation set.

Pheromone trail update: Update the pheromone trail on each edge based on the quality of the solution found by the ant. The stronger the performance of the model, the stronger the reinforcement of the pheromone trail. Conversely, if the performance is poor, the pheromone trail is weakened or evaporated.

Termination: Repeat steps 2–4 for a fixed number of iterations or until a termination criterion is met. The subset of features with the strongest pheromone trail after the final iteration is returned as the final solution.

By updating the pheromone trail based on the quality of the solution, the ACO algorithm effectively searches the feature space and learns which features are most important for the given task. The features with the strongest pheromone trail correspond to the ones that are most frequently selected by the ants, and thus, are the most important ones for the task at hand.

### 3.6. Inductive and Deductive Phase

The classification stage is the last stage of medical image processing. The accuracy at this stage depends on the efficiency in the previous stages. After image optimization and extraction of malignant lymphocytes by the segmentation algorithm, malignant lymphocytes were analyzed by the deep learning models to extract high-dimensional feature maps. The ACO algorithm reduces the dimensions to select the best features and eliminate redundant features. The XGBoost and TD networks receive the critical features, train them, and validate them with 80% to build a classification model called the inductive phase. In addition, 20% of the data were randomly isolated to test the system’s performance, called the deductive phase.

XGBoost and DT are popular machine learning methods that have different strengths and characteristics, which make them suitable for different scenarios. The reason for choosing XGBoost and DT as specific machine learning methods depends on the context and requirements of the problem at hand. Here are some explanations for their selection:

XGBoost:

XGBoost is an optimized implementation of the gradient boosting framework, which is an ensemble learning technique. It combines multiple weak predictive models (typically decision trees) to create a more accurate and robust model. XGBoost offers several advantages:

- High performance: XGBoost is known for its scalability and efficiency. It has been optimized to handle large datasets with millions of observations and thousands of features. It can also take advantage of parallel computing, making it suitable for high-performance computing environments.
- Regularization techniques: XGBoost includes regularization techniques such as L1 and L2 regularization, which help to prevent overfitting and improve generalization.
- Feature importance: XGBoost provides a measure of feature importance, which helps in identifying the most influential features for making predictions. This can be valuable for feature selection and understanding the underlying patterns in the data.
- Flexibility: XGBoost supports a wide range of loss functions, making it adaptable to various problem types, including regression, classification, and ranking tasks.

Decision Trees:

Decision trees are a simple yet powerful machine learning technique that builds a tree-like model of decisions and their possible consequences. Each internal node represents a decision based on a feature, and each leaf node represents a prediction or a class label. Decision trees have several advantages:

- Interpretability: Decision trees are easy to understand and interpret. The generated tree structure allows humans to follow the decision-making process and gain insights into how the model arrives at its predictions.
- Handling non-linear relationships: Decision trees can capture non-linear relationships between features and the target variable. They can handle both continuous and categorical features without requiring extensive preprocessing.
- Feature interactions: Decision trees can capture interactions between features, which can be essential for modeling complex relationships in the data.
- Outlier robustness: Decision trees are less sensitive to outliers compared to some other machine learning methods. They partition the feature space into regions and make predictions based on the majority class within each region.

Overall, XGBoost and decision trees are chosen based on their specific strengths and the requirements of the problem. XGBoost is often favored when high performance, scalability, and improved predictive accuracy are desired. Decision trees, on the other hand, are selected when interpretability, handling non-linear relationships, and capturing feature interactions are important considerations.

#### 3.6.1. XGBoost Algorithm

XGBoost (extreme gradient boosting) is an extension of the gradient boosting method, combining many weak learners’ outputs to create a strong learner. The XGBoost algorithm involves the construction of a sequence of decision trees, where each tree is trained on the residuals of the previous tree. The output of the final tree is the prediction of the XGBoost model. The XGBoost algorithm minimizes a loss function, a combination of a regularization term, and a differentiable loss function. The differentiable loss function can be any function that measures the difference of the predicted and the true output. XGBoost uses gradient boosting to optimize the objective function [42]. The gradient of the loss function concerning the model’s predicted output is computed, and the output of the next tree is trained to minimize the gradient. XGBoost also uses a technique called “gradient-based sampling” to speed up the training process. During the construction of each tree, XGBoost uses the gradient of the loss function to prioritize the samples that are most important for improving the model. This helps to reduce the amount of time needed to train the model. The XGBoost algorithm receives the fused features of the MobileNet, VGG16, and AlexNet models and also receives the features of the MobileNet, VGG16, and AlexNet models without merging them. XGBoost trains the feature matrix and divides it into training, validation, and performance testing.

#### 3.6.2. Decision Tree Algorithm

A decision tree algorithm is a machine learning algorithm commonly used for classification tasks. The algorithm creates a tree-like model of decisions and their possible results. The tree consists of nodes, branches, and leaves. The nodes represent decisions based on input features, the branches represent the possible outcomes of those decisions, and the leaves represent the final predictions or classifications. Each decision is made based on the most informative feature or combination of features that can best split the data into groups or categories. During the training process, the DL method recursively partitions the feature into subsets based on the feature values, and it chooses the best feature to split the data at each node [43]. The process continues until a stopping measure is met, such as all instances at a node belonging to the same class or when a maximum depth of the tree is reached. The DT algorithm receives the fused features of the MobileNet, VGG16, and AlexNet models and also receives the features of the MobileNet, VGG16, and AlexNet models without merging them. The DT method trains the feature matrix and divides it into training, validation, and performance testing.

Let us discuss the complexity of the XGBoost and DT algorithms and the computational considerations associated with these approaches.

XGBoost:

XGBoost is an ensemble learning algorithm that combines the predictions of multiple weak classifiers, typically decision trees, to form a strong classifier. It is known for its speed and high performance. The complexity of XGBoost can be divided into two main aspects:

- Training Complexity: The training process of XGBoost involves constructing decision trees iteratively. Each tree is built to correct the mistakes made by previous trees. The complexity of training an XGBoost model depends on the following factors:

Number of trees: The more trees you have, the higher the training complexity.

Maximum tree depth: A deeper tree can capture more complex patterns but also increases training time.

Number of features: Higher-dimensional feature spaces can lead to longer training times.

Regularization parameters: XGBoost has regularization parameters to control overfitting. Adjusting these parameters can affect training time.

b. Prediction Complexity: Once the XGBoost model is trained, making predictions for new samples is generally fast. The complexity depends on the number of trees in the model, the maximum depth of the trees, and the number of features.

Overall, XGBoost is computationally efficient due to its parallelization capabilities and various optimization techniques. It can handle large datasets and high-dimensional feature spaces effectively. However, the training time may still depend on the specific dataset size and complexity.

Decision Trees:

Decision trees are a fundamental component of XGBoost, and they can also be used as standalone classifiers. The complexity of decision trees is primarily determined by the following factors:

- Training Complexity: Building a decision tree involves recursively partitioning the feature space based on the selected splitting criteria, such as Gini impurity or information gain. The training complexity of a decision tree depends on:

Number of samples: More samples require more time to evaluate potential splits.

Number of features: Higher-dimensional feature spaces increase the search space for optimal splits.

Stopping criteria: Early stopping rules, such as maximum tree depth or minimum sample requirements, can reduce complexity but may affect accuracy.

b. Prediction Complexity: Once the decision tree is constructed, making predictions for new samples is efficient. Traversing the tree and evaluating the corresponding features have a complexity proportional to the tree’s depth.

Decision trees are interpretable, easy to understand, and can handle categorical and numerical features. However, they may suffer from overfitting on complex datasets and lack the ability to capture more intricate patterns compared to ensemble methods such as XGBoost.

In the context of fusing features between two CNN models with handcrafted features for detecting malignant lymphocytes, the performance and feasibility of XGBoost and DT depend on factors such as the features’ quality and diversity, the dataset’s size, and the problem’s complexity. By combining the strengths of CNNs and handcrafted features with the ensemble learning capabilities of XGBoost, you can potentially achieve improved accuracy and generalization.

### 3.7. Strategy of Implementation Sequence

In this study, several strategies were applied. We review the steps of the systems’ implementation sequence to evaluate the malignant lymphoma dataset.

#### 3.7.1. Hybrid Strategy of Machine Learning with Features of DL Models

**Figure 3:**
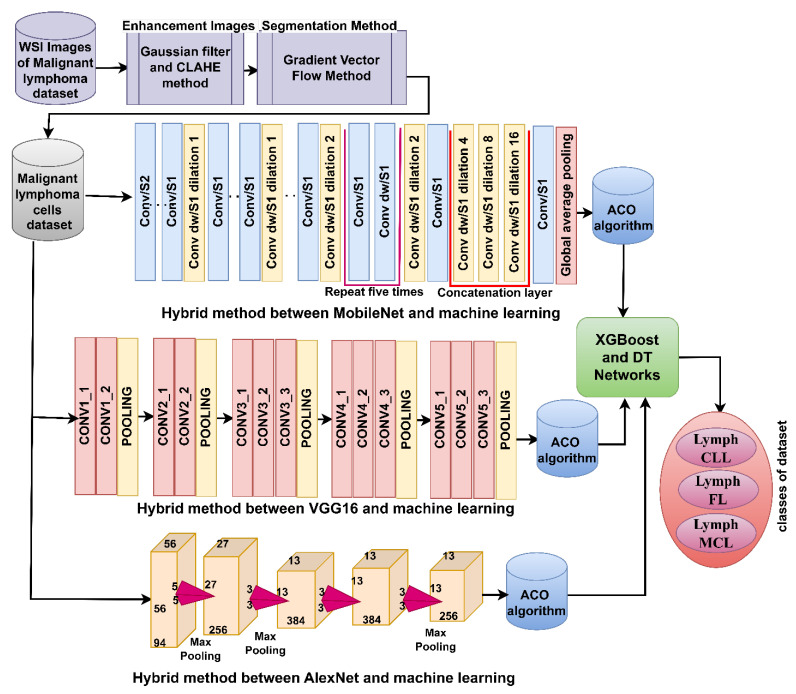
Analysis of WSI images for the diagnosis of malignant lymphomas by the XGBoost and DT networks based on the features of DL models.

This strategy for analyzing the WSI image of malignant lymphomas goes through the following steps [44], as shown in Figure 3 [45]. First, image optimization by Gaussian and Laplacian filters. Second, extracting malignant lymphocytes and isolating them from the rest of the image by the GVF method. Third, feeding malignant lymphoma cells into DL models to extract high-dimensional deep feature maps. Fourth, reducing the high-dimensional feature maps, selecting the most important features using the ACO method, and saving them at sizes of 15,000 × 525, 15,000 × 890, and 15,000 × 910 for the MobileNet, VGG16, and AlexNet models, respectively. Fifth, feeding the selected features to the XGBoost and DT networks.

#### 3.7.2. Hybrid Strategy of Machine Learning with Fusion Features of DL Models

**Figure 4:**
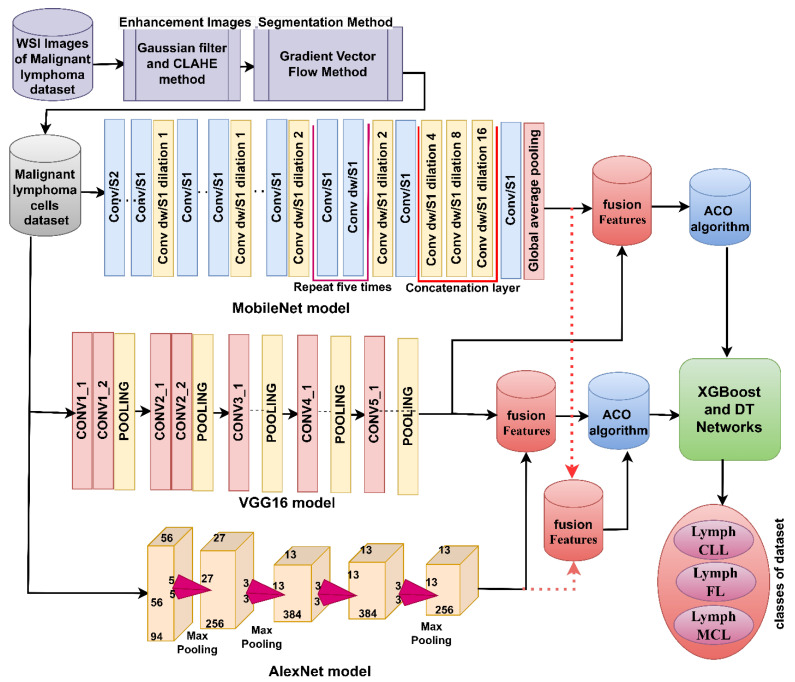
Analysis of WSI images for the diagnosis of malignant lymphomas by the XGBoost and DT networks based on the fused features of DL models.

This strategy for analyzing the WSI image of malignant lymphomas goes through the following steps, as illustrated in Figure 4. First, image optimization by Gaussian and Laplacian filters. Second, extraction of the malignant lymphocytes and isolation of them from the rest of the image using the GVF method. Third, feeding the malignant lymphocytes into DL models to extract high-dimensional deep feature maps. Fourth, the MobileNet, VGG16, and AlexNet model feature maps are merged sequentially as follows: MobileNet-VGG16, VGG16-AlexNet, and MobileNet-AlexNet [46]. Fifth, reducing the high-dimensional feature maps, selecting the essential features using the ACO method, and saving them at sizes of 15,000 × 720, 15,000 × 1030, and 15,000 × 1120 for the fusion models of MobileNet-VGG16, VGG16-AlexNet, and MobileNet-AlexNet, respectively. Sixth, feeding the specific features to the XGBoost and DT networks.

#### 3.7.3. Hybrid Strategy of Machine Learning with Fusion Features of DL and Handcrafted

The parameter values used to extract the specified features may vary depending on the specific implementation and the dataset used.

Fuzzy Color Histogram (FCH):

The FCH algorithm extracts color information from an image. The number of features extracted, in this case, is 16. The specific parameter values depend on factors such as the color space used (e.g., RGB, HSV, Lab) and the number of bins used to discretize the color space. For example, if a 3D color histogram with 4 bins per channel is used, it would result in 4 × 4 × 4 = 64 bins. However, since the mentioned number of features is 16, it is possible that some additional processing or feature selection techniques were applied to reduce the dimensionality of the histogram representation.

Discrete Wavelet Transform (DWT):

DWT is a signal processing technique for analyzing signals in different frequency bands. The number of features extracted using DWT in this case is 12. The specific parameter values would depend on the wavelet used, the level of decomposition, and the choice of approximation and detail coefficients. Typically, the DWT is performed by decomposing the image into multiple levels, resulting in a set of approximate and detailed coefficients. The number of features could be determined by selecting a subset of these coefficients or by using certain statistical measures on the coefficients, such as mean or standard deviation.

Local Binary Patterns (LBPs):

An LBP is a texture descriptor that captures the local structure of an image. The number of features extracted using the LBP is 203. The LBP operates by comparing the intensity values of pixels in a neighborhood around each pixel and encoding the results into a binary pattern. The number of features can vary based on factors such as the size of the neighborhood, the number of sampling points within the neighborhood, and the specific encoding scheme used.

Gray-Level Co-occurrence Matrix (GLCM):

GLCM is a texture analysis method that characterizes the spatial relationship between pixel intensity values. The number of features extracted using GLCM is 24. The parameter values for GLCM typically include the distance and angle between the pixel pairs considered, as well as the number of gray levels to quantize the image. The resulting GLCM is then computed for each pixel pair, and various statistical measures (e.g., contrast, energy, entropy) are calculated from the matrix. The specific combinations of distance, angle, and gray level quantization are chosen to extract 24 features that capture different aspects of texture information.

The reason behind using a relatively large number of features in this case could be to capture a wide range of visual characteristics from the images. By extracting multiple types of features, such as color, texture, and wavelet coefficients, the aim is to capture diverse aspects of the image content that are relevant for their specific application. Having a rich and diverse set of features can potentially improve the performance of subsequent classification or analysis tasks by providing a more comprehensive representation of the data. However, it is important to note that the choice of feature extraction techniques and the number of features depend on the specific application and dataset, and it is possible to use feature selection or dimensionality reduction techniques to further refine the feature set.

**Figure 5:**
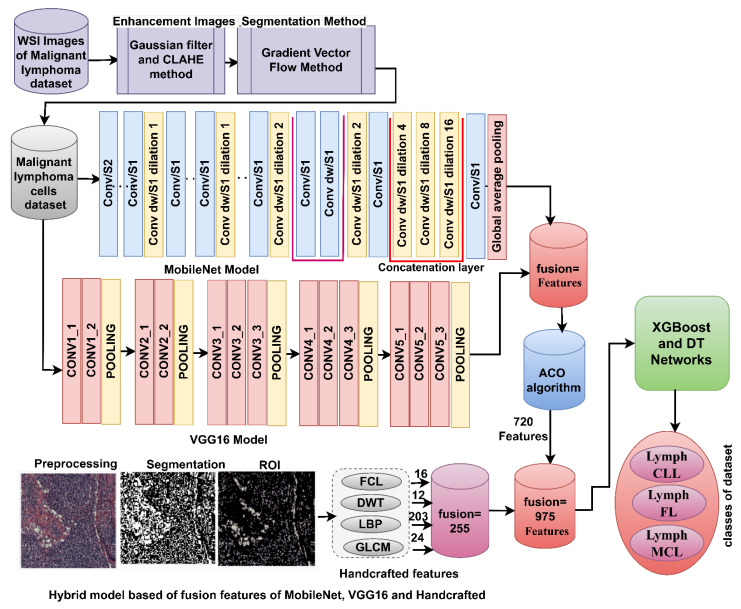
Analysis of WSI images for the diagnosis of malignant lymphomas by the XGBoost and DT networks based on fused features between DL models and handcrafted features.

This strategy for WSI image analysis of malignant lymphomas goes through the following steps. The first five implementation phases of the previous strategic phase are the same as for this strategy. Sixth, the FCH, DWT, LBP, and GLCM algorithms receive malignant lymphoma cells to extract the handcrafted features [47]: a total of 16 color features are extracted using the FCH algorithm; the DWT algorithm extracts 12 features from each image; the LBP algorithm extracts 203 bi-surface texture features; the GLCM algorithm extracts 24 texture features. Seventh, the features of the FCH, DWT, LBP, and GLCM algorithms are merged into feature vectors with a size of 255 features, so the size of the dataset features is 15,000 × 255. Eighth, the fusion features of the MobileNet-VGG16 and VGG16-AlexNet models are merged with the handcrafted features. Thus, the datasets’ features become 15,000 × 975 and 15,000 × 1285. Ninth, the distinguishing features are fed to the XGBoost and DT networks to classify them with high efficiency. Figure 5 shows the analysis of WSI images for the diagnosis of malignant lymphomas by XGBoost and DT based on MobileNet, VGG16, and handcrafted features.

## 4. Results of System Performance

### 4.1. Evaluation Metrics

There are many tools for measuring the performance of systems, and the confusion matrix is considered one of the most important system measures. Still, it is the gold standard for evaluating systems. A confusion matrix is a form of a quadrilateral table in which the number of columns equals the number of rows. The confusion matrix contains the number of images of the test dataset, where each class is represented by a column. All of the samples are classified by the system correctly in the main diameter, which is called TP, while the other cells of the matrix represent the actual images that belong to a class, while the system predicted them incorrectly in other classes, called FP and FN. Equations (11)–(15) show the system evaluation measures. Note the variables TP, TN, FP, and FN, which are supplied from the confusion matrix.

(11) $$AUC=\frac{TPRate}{FPRate}*100\%$$

(12) $$Accuracy=\frac{TN+TP}{TN+TP+FN+FP}*100\%$$

(13) $$Precision=\frac{TP}{TP+FP}*100\%$$

(14) $$Sensitivity=\frac{TP}{TP+FN}*100\%$$

(15) $$Specificity=\frac{TN}{TN+FP}*100$$

### 4.2. Results of Pre-trained Networks

This section discusses the performance of the MobileNet, VGG16, and AlexNet models pre-trained on WSI images from the malignant lymphoma dataset. In these models, the knowledge gained from these models, when trained on the ImageNet dataset, was transferred to perform new tasks to create a new dataset. WSI images of malignant lymphomas were fed into DL models and analyzed through convolutional and adjuvant layers. Classified through fully connected layers, the activation function sorts each image into its correct class.

Table 2 summarizes the performance of the MobileNet, VGG16, and AlexNet models pre-trained on WSI images for the malignant lymphoma dataset. MobileNet achieved an AUC of 93.63%, accuracy of 92.7%, precision of 92.73%, sensitivity of 92.8%, and specificity of 96.6%. While VGG16 yielded an AUC of 92.8%, accuracy of 92.3%, precision of 92.37%, sensitivity of 92.4%, and specificity of 96.47%. AlexNet obtains an AUC of 92.5%, accuracy of 91.9%, precision of 91.87%, sensitivity of 91.83%, and specificity of 95.93%.

### 4.3. Results of Hybrid Strategy of Machine Learning with Features of DL Models

This section discusses the performance of the hybrid networks of DL models (MobileNet, VGG16, and AlexNet) with the XGBoost and DT networks for diagnosing malignant lymphomas. The images were enhanced, and malignant lymphocytes were extracted and isolated from the rest of the image using the GVF algorithm. This technique works in two parts: the first part is the DL networks extract deep feature maps from malignant lymphoma cells, applying the ACO algorithm to select important features. The second part is the XGBoost and DT networks are used for classifying important feature maps.

Table 3 summarizes the results of the XGBoost network with features of the MobileNet, VGG16, and AlexNet models for diagnosing WSI images of malignant lymphomas. The MobileNet-XGBoost network resulted in an AUC of 97.4%, accuracy of 96.7%, precision of 96.7%, sensitivity of 96.87%, and specificity of 98.23%. While the VGG16-XGBoost network resulted in an AUC of 96.23%, accuracy of 96.4%, precision of 96.37%, sensitivity of 96.3%, and specificity of 97.83%. While the AlexNet-XGBoost network yielded an AUC of 96.6%, accuracy of 95.8%, precision of 95.83%, sensitivity of 95.87%, and specificity of 97.87%.

The XGBoost network with features of MobileNet, VGG16, and AlexNet produces a confusion matrix, which shows the performance of these hybrid networks for diagnosing malignant lymphomas.

**Figure 6:**
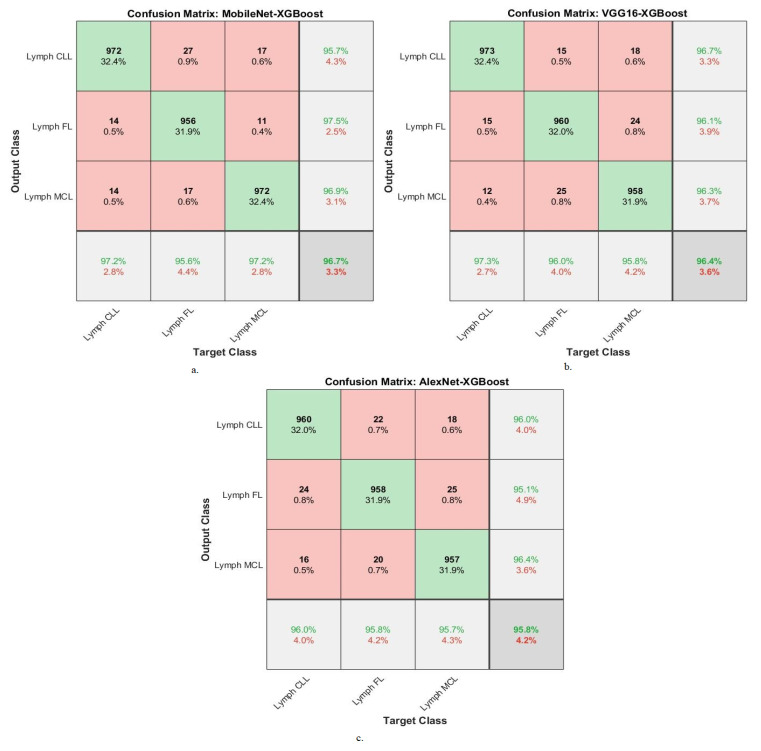
Confusion matrix showing performance results of DL-XGBoost hybrid models based on the ACO method for the diagnosis of malignant lymphomas: (**a**). MobileNet-XGBoost; (**b**). VGG16-XGBoost; (**c**). AlexNet-XGBoost.

Figure 6 shows the confusion matrix for the MobileNet-XGBoost, VGG16-XGBoost, and AlexNet-XGBoost technologies. The figures show the accuracy of each technique for each class of the dataset. MobileNet-XGBoost achieved the following accuracy for each type of malignant lymphoma: for the lymph CLL class of 97.2%, for the lymph FL class of 95.6%, and for the lymph MCL class of 97.2%. VGG16-XGBoost achieved the following accuracy for each type of malignant lymphoma: for the lymph CLL class of 97.3%, for the lymph FL class of 96%, and for the lymph MCL class of 95.6%. AlexNet-XGBoost achieved the following accuracy for each type of malignant lymphoma: for the lymph CLL class of 96%, for the lymph FL class of 95.8%, and for the lymph MCL class of 95.7%.

Table 4 summarizes the results of the DT network with features of the MobileNet, VGG16, and AlexNet models for diagnosing WSI images of malignant lymphomas. The MobileNet-DT network resulted in an AUC of 95.83%, accuracy of 94.8%, precision of 94.73%, sensitivity of 94.93%, and specificity of 97.37%. While the VGG16-DT network resulted in an AUC of 95.77%, accuracy of 94.4%, precision of 94.43%, sensitivity of 94.37%, and specificity of 97.17%. While the AlexNet-DT network yielded an AUC of 95.53%, accuracy of 93.8%, precision of 93.63%, sensitivity of 93.57%, and specificity of 97.1%.

The DT network with features of MobileNet, VGG16, and AlexNet produces a confusion matrix, which shows the performance of these hybrid networks for diagnosing malignant lymphomas.

**Figure 7:**
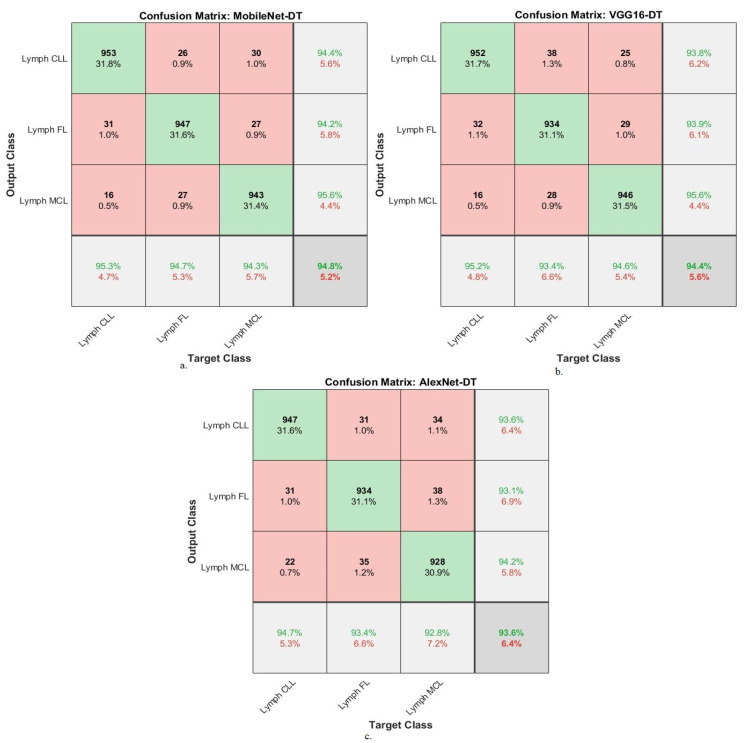
Confusion matrix showing performance results of DL-DT hybrid models based on the ACO method for the diagnosis of malignant lymphomas. (**a**). MobileNet-DT, (**b**). VGG16-DT, (**c**). AlexNet-DT.

Figure 7 shows the confusion matrix for the MobileNet-DT, VGG16-DT, and AlexNet-DT technologies. The matrices show the accuracy of each technique for each class of the dataset. MobileNet-DT achieved the following accuracy for each type of malignant lymphoma: for the lymph CLL class of 95.3%, for the lymph FL class of 94.7%, and for the lymph MCL class of 94.3%. VGG16-DT achieved the following accuracy for each type of malignant lymphoma: for the lymph CLL class of 95.2%, for the lymph FL class of 93.4%, and for the lymph MCL class of 94.6%. AlexNet-DT achieved the following accuracy for each type of malignant lymphoma: for the lymph CLL class of 94.7%, for the lymph FL class of 93.4%, and for the lymph MCL class of 92.8%.

### 4.4. Result of Hybrid Strategy of Machine Learning with Fusion Features of DL Models

This section discusses the performance of the XGBoost and DT algorithms with the fused features of the DL networks for diagnosing malignant lymphomas. The images were optimized, and malignant lymphocytes were extracted and isolated from the rest of the image using the GVF algorithm. This technique works in two parts: the first is extracting features from DL networks to extract deep feature maps from malignant lymphoma cells. Features of the DL networks were serially integrated into feature vectors in the following order: MobileNet-VGG16, VGG16-AlexNet, and MobileNet-AlexNet. High-dimensional feature vectors were sent to the ACO algorithm to identify important features and delete redundant ones. In the second part, the XGBoost and DT networks were used for classifying the WSI images represented by feature vectors.

The importance of applying overlapping filters is clearly very important, as it was noted that the results improved significantly when applying the Gaussian filter and then the Laplacian filter. When a Gaussian filter was applied, XGBoost with the features of Mobile-Net-VGG16 achieved an AUC of 94.21%, accuracy of 93.41%, precision of 93.89%, sensitivity of 94.78%, and specificity of 96.1%. However, it is noted from the table below that the results are better when applying the Gaussian and Laplacian filters.

Table 5 summarizes the results of the XGBoost network with combined features of the MobileNet-VGG16, VGG16-AlexNet, and MobileNet-AlexNet models for diagnosing WSI images of malignant lymphomas. XGBoost with MobileNet-VGG16 features achieved an AUC of 98.27%, accuracy of 98.5%, precision of 98.5%, sensitivity of 98.4%, and specificity of 98.8%. While the XGBoost with VGG16-AlexNet features resulted in an AUC of 98.3%, accuracy of 98.4%, precision of 98.37%, sensitivity of 98.4%, and specificity of 98.87%. While the XGBoost with MobileNet-AlexNet features yielded an AUC of 97.8%, accuracy of 98.2%, precision of 98.23%, sensitivity of 98.27%, and specificity of 98.93%.

**Figure 8:**
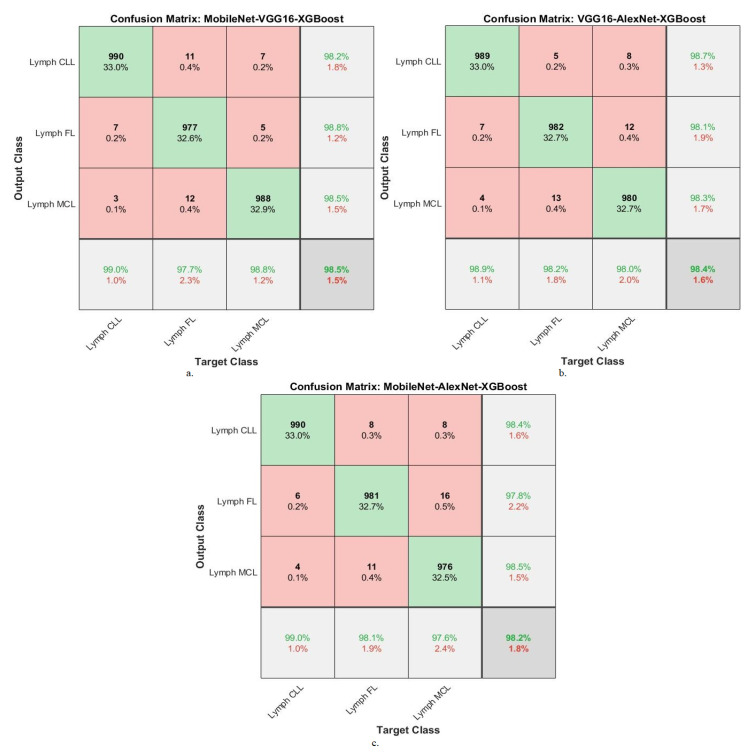
Confusion matrix showing performance results of XGBoost with hybrid features of DL models based on the ACO method for diagnosing malignant lymphomas. (**a**). MobileNet-VGG16-XGBoost, (**b**). VGG16-XAlexNet-XGBoost, (**c**). MobileNet-AlexNet-XGBoost.

Figure 8 shows the confusion matrix for the MobileNet-VGG16-XGBoost, VGG16-AlexNet-XGBoost, and MobileNet-AlexNet-XGBoost technologies. The figures show the accuracy of each technique for each class of the dataset. MobileNet-VGG16-XGBoost achieved the following accuracy for each type of malignant lymphoma: for the lymph CLL class of 99%, for the lymph FL class of 97.7%, and for the lymph MCL class of 98.8%. VGG16-AlexNet-XGBoost achieved the following accuracy for each type of malignant lymphoma: for the lymph CLL class of 98.9%, for the lymph FL class of 98.2%, and for the lymph MCL class of 98%. MobileNet-AlexNet-XGBoost achieved the following accuracy for each type of malignant lymphoma: for the lymph CLL class of 99%, for the lymph FL class of 98.1%, and for the lymph MCL class of 97.6%.

Table 6 summarizes the results of the DT network with combined features of the MobileNet-VGG16, VGG16-AlexNet and MobileNet-AlexNet models for diagnosing WSI images of malignant lymphomas. DT with MobileNet-VGG16 features achieved an AUC of 98.1%, accuracy of 97.8%, precision of 97.8%, sensitivity of 97.87%, and specificity of 98.8%. While the DT with VGG16-AlexNet features resulted in an AUC of 98.13%, accuracy of 97.7%, precision of 97.67%, sensitivity of 97.7%, and specificity of 98.97%. While the DT with MobileNet-AlexNet features yielded an AUC of 98.4%, accuracy of 97.3%, precision of 97.33%, sensitivity of 96.83%, and specificity of 98.47%.

**Figure 9:**
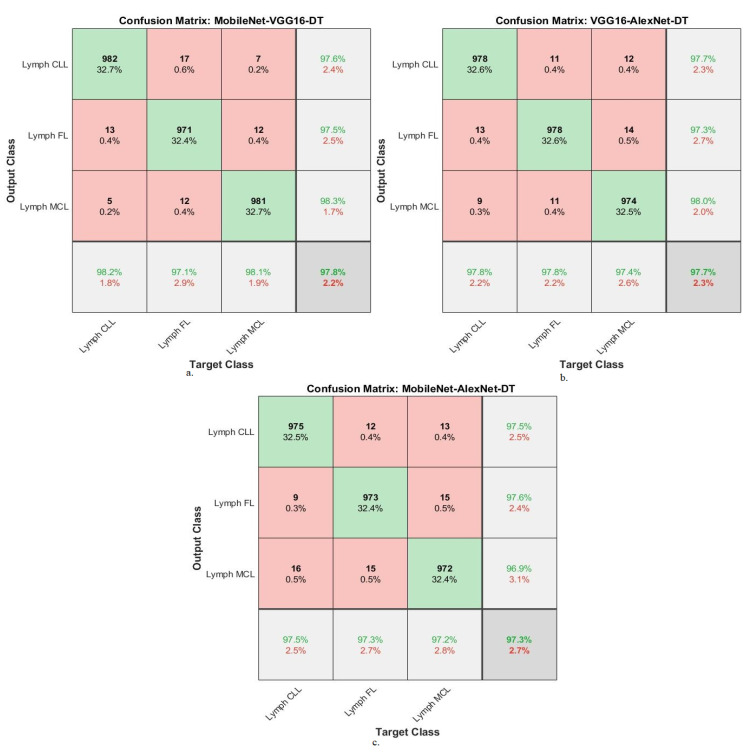
Confusion matrix showing performance results of DT with hybrid features of DL models based on the ACO method for diagnosing malignant lymphomas. (**a**). MobileNet-VGG16-DT, (**b**). VGG16-XAlexNet-DT, (**c**). MobileNet-AlexNet-DT.

Figure 9 shows the confusion matrix for MobileNet-VGG16-DT, VGG16-AlexNet-DT, and MobileNet-AlexNet-DT technologies. The figures show the accuracy of each technique for each class of the dataset. MobileNet-VGG16-DT achieved the following accuracy for each type of malignant lymphoma: for the lymph CLL class of 98.2%, for the lymph FL class of 97.1%, and for the lymph MCL class of 98.1%. VGG16-AlexNet-DT achieved the following accuracy for each type of malignant lymphoma: for the lymph CLL class of 97.8%, for the lymph FL class of 97.8%, and for the lymph MCL class of 97.4%. MobileNet-AlexNet-DT achieved the following accuracy for each type of malignant lymphoma: for the lymph CLL class of 97.5%, for the lymph FL class of 97.3%, and for the lymph MCL class of 97.2%.

### 4.5. Results of Machine Learning with Fusion Features of DL and Handcrafted

The section discusses the performance of the the XGBoost and DT algorithms with the fused features of the DL networks and handcrafted features for diagnosing malignant lymphomas. The images were optimized, and malignant lymphocytes were extracted and isolated from the rest of the image by the GVF algorithm. This technique works in two parts: the first is extracting features from the DL networks to extract deep feature maps from malignant lymphoma cells. The features of the DL networks were serially integrated into feature vectors in the following order: MobileNet-VGG16, VGG16-AlexNet, and MobileNet-AlexNet. Then, high-dimensional feature vectors were sent to the ACO algorithm to identify important features and delete redundant ones. The handcrafted features from the traditional methods were then extracted and combined. Then, the features of the DL models were combined with the features of the handcrafted models. In the second part, the XGBoost and DT networks for classifying WSI images represented by feature vectors were used.

Table 7 summarizes the results of the XGBoost network with combined features of the MobileNet-VGG16-handcrafted, VGG16-AlexNet-handcrafted, and MobileNet-AlexNet-handcrafted models for diagnosing WSI images of malignant lymphomas. XGBoost with MobileNet-VGG16-handcrafted features achieved an AUC of 99.43%, accuracy of 99.8%, precision of 99.77%, sensitivity of 99.7%, and specificity of 99.8%. While XGBoost with VGG16-AlexNet-handcrafted features resulted in an AUC of 99.57%, accuracy of 99.7%, precision of 99.73%, sensitivity of 99.67%, and specificity of 99.7%. While XGBoost with MobileNet-AlexNet-handcrafted features yielded an AUC of 99.4%, accuracy of 99.5%, precision of 99.5%, sensitivity of 99.43%, and specificity of 99.7%.

**Figure 10:**
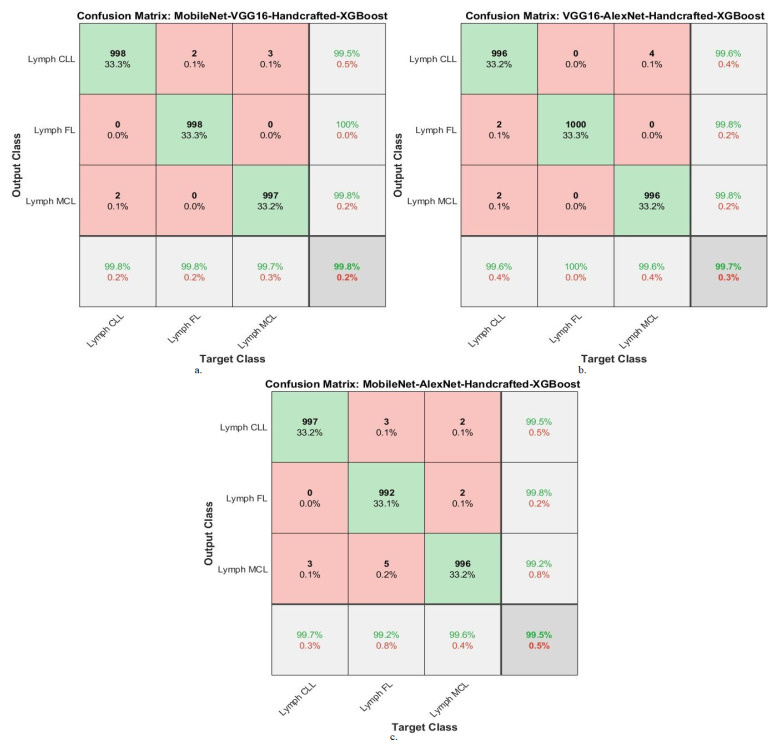
Confusion matrix showing performance results of XGBoost with hybrid features of DL and handcrafted features based on the ACO method for diagnosing malignant lymphomas. (**a**). MobileNet-VGG16-Handcrafted-XGBoost, (**b**). VGG16-XAlexNet-Handcrafted-XGBoost, (**c**). MobileNet-AlexNet-Handcrafted-XGBoost.

Figure 10 confusion matrix for XGBoost with features of MobileNet-VGG16-Handcrafted, VGG16-AlexNet-Handcrafted and MobileNet-AlexNet-Handcrafted technologies. The figures show the accuracy of each technique for each class of the dataset. The XGBoost network with features of MobileNet-VGG16-Handcrafted achieved the following accuracy for each type of malignant lymphoma: for the lymph CLL class of 99.8%, for the lymph FL class of 99.8%, and for the lymph MCL class of 99.7%. The XGBoost network with features of VGG16-AlexNet-Handcrafted achieved the following accuracy for each type of malignant lymphoma: for the lymph CLL class of 99.6%, for the lymph FL class of 100%, and for the lymph MCL class of 99.6%. The XGBoost network with features of MobileNet-AlexNet-Handcrafted achieved the following accuracy for each type of malignant lymphoma: for the lymph CLL class of 99.7%, for the lymph FL class of 99.2%, and for the lymph MCL class of 99.6%.

Table 8 summarizes the results of the DT network with combined features of the MobileNet-VGG16-handcrafted, VGG16-AlexNet-handcrafted, and MobileNet-AlexNet-handcrafted models for diagnosing WSI images of malignant lymphomas. The DT with MobileNet-VGG16-handcrafted features achieved an AUC of 99.37%, accuracy of 99.3%, precision of 99.27%, sensitivity of 99.03%, and specificity of 99.67%. While the DT with VGG16-AlexNet-handcrafted features resulted in an AUC of 98.53%, accuracy of 98.7%, precision of 98.73%, sensitivity of 99%, and specificity of 99.2%. While the DT with MobileNet-AlexNet-handcrafted features yielded an AUC of 99.03%, accuracy of 98.8%, precision of 99.77%, sensitivity of 99.07%, and specificity of 99.13%.

**Figure 11:**
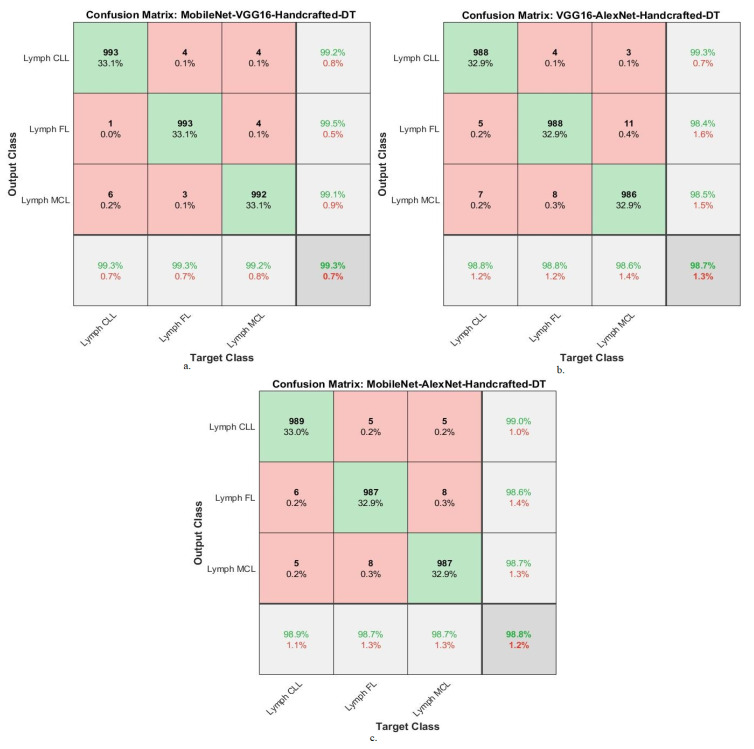
Confusion matrix showing performance results of DT with hybrid features of DL and handcrafted features based on the ACO method for diagnosing malignant lymphomas. (**a**). MobileNet-VGG16-Handcrafted-DT, (**b**). VGG16-AlexNet-Handcrafted-DT, (**c**). MobileNet-AlexNet-Handcrafted-DT.

Figure 11 confusion matrix for DT with features of MobileNet-VGG16-Handcrafted, VGG16-AlexNet-Handcrafted, and MobileNet-AlexNet-Handcrafted technologies. The figures show the accuracy of each technique for each class of the dataset. The DT network with features of MobileNet-VGG16-Handcrafted achieved the following accuracy for each type of malignant lymphoma: for the lymph CLL class of 99.3%, for the lymph FL class of 99.3%, and for the lymph MCL class of 99.2%. The DT network with features of VGG16-AlexNet-Handcrafted achieved the following accuracy for each type of malignant lymphoma: for the lymph CLL class of 98.8%, for the lymph FL class of 98.8%, and for the lymph MCL class of 98.6%. The DT network with features of MobileNet-AlexNet-Handcrafted achieved the following accuracy for each type of malignant lymphoma: for the lymph CLL class of 98.9%, for the lymph FL class of 98.7%, and for the lymph MCL class of 98.8%.

## 5. Discussion of Systems’ Performance

Malignant lymphoma is a disease that leads to death due to the exposure of lymphocytes to malignant tumors. Biopsies taken from the patient are the gold standard for analysis. The biopsy is placed on a slide stained with hematoxylin and eosin (H&E) for analysis. However, manual diagnostics is still error-prone and has many limitations. Thus, there is a great need for automated diagnostics using AI techniques that address the limitations of manual diagnosis. Several investigators have developed techniques for diagnosing WSI images of malignant lymphomas. The techniques used by the researchers in their previous studies and findings were reviewed. It is noted from previous studies that gaps hindered the achievement of high accuracy due to the similarity of characteristics between types of lymphomas.

This work diagnosed images of malignant lymphomas with various techniques and tools. The first strategy was to analyze images of malignant lymphomas using pre-trained MobileNet, VGG16, and AlexNet networks. MobileNet achieved 92.7% accuracy, VGG16 achieved 92.3% accuracy, and AlexNet achieved 91.9% accuracy.

The second strategy adopted was to analyze images of malignant lymphomas using the XGBoost and DT networks when fed separately with the features of the MobileNet, VGG16, and AlexNet models. XGBoost with the MobileNet, VGG16, and AlexNet features achieved an accuracy of 96.7%, 96.4%, and 95.8%, respectively. While the DL network with the features of MobileNet, VGG16, and AlexNet resulted in an accuracy of 94.8%, 94.4%, and 93.8%, respectively.

The third strategy for analyzing malignant lymphoma images was based on integrating the characteristics of DL networks (MobileNet-VGG16, VGG16-AlexNet, and MobileNet-AlexNet) and classifying them using the XGBoost and DT networks. XGBoost with MobileNet-VGG16’s features achieved 98.5% accuracy, while with the features of VGG16-AlexNet, it achieved 98.4% accuracy, while when fed with MobileNet-AlexNet’s features, it achieved 98.2% accuracy. While the DL network with MobileNet-VGG16’s features achieved 97.8% accuracy, while with the features of VGG16-AlexNet, it achieved 97.7% accuracy. When the XGBoost network was fed with MobileNet-AlexNet’s features, it achieved 97.3% accuracy.

The fourth strategy for analyzing malignant lymphoma images was based on integrating the features of DL networks (MobileNet-VGG16, VGG16-AlexNet, and MobileNet-AlexNet) with the handcrafted features classified by the XGBoost and DT networks. The XGBoost network with both features of MobileNet-VGG16 and handcrafted features achieved 99.8% accuracy, while with both features of VGG16-AlexNet and handcrafted features, it achieved 99.7% accuracy. When fed with both features of MobileNet-AlexNet and handcrafted features, it achieved 99.5% accuracy. While the DL network with both features of MobileNet-VGG16 and handcrafted features achieved 99.3% accuracy, while with both features of VGG16-AlexNet and handcrafted features, it achieved 98.7% accuracy. When the DL network was fed with both features of MobileNet-AlexNet and handcrafted features, it reached 98.8% accuracy.

It is noted that the results improved when separating the lymphoma cells from the rest of the image using the GVF algorithm and applying the ACO algorithm to select the features. The improvement of the performance of the hybrid systems between the XGBoost-DL and DT-DL networks is better than the performance of the pre-trained models. At the same time, the results improved significantly when DL features were combined and classified by the XGBoost and DT networks. Finally, when combining texture, color, and shape features (handcrafted features) with the fusion features of the DL networks, the XGBoost and DT networks achieve superior results compared to other methods. We conclude that the combination of the handcrafted features with the features of the DL networks represents the characteristics of the images with a high representation, which results in the XGBoost and DT networks’ superior results.

The provided information includes summaries of various studies related to the diagnosis and analysis of lymphomas using different methods and techniques. There follows a comparison of the methods and results of previous studies. Irshaid et al. [11] trained CNNs on WSI smear images to predict lymphoma progression. Extracted features and morphological parameters were used. They achieved an AUC of 92.3%. Xia et al. [12] constructed a CNN using various image maps to diagnose lymphoma. It achieved an accuracy of 88.4%, sensitivity of 93.4%, and AUC of 95.6%. Savas et al. [13] used a CNN-LSTM method on augmented WSI images. It achieved an accuracy of 96.65%, sensitivity of 96.51%, and F1 score of 96.67%. Miyoshi et al. [14] utilized deep learning networks for image classification of histological WSI images. They achieved an accuracy of 94% with 5× magnification and 92% with 40× magnification. Zhang et al. [17] analyzed WSI images of malignant lymph nodes using a backpropagation (BP) network and genetic algorithm (GA) optimization. They achieved an accuracy of 96% with BP and 97.7% with GA-BP. Zahra et al. [19] explored deep learning methods for diagnosing histological images with different magnification factors. MobileNet achieved 53% accuracy with 100× magnification, and Inception-V3 achieved 80% accuracy with 400× magnification. Zhang et al. [20] trained a CNN model on lymph node regions using convolutional, dropout, max pooling, and dense layers. This achieved an accuracy of 93.17% compared to 84.49% for the AlexNet model. Swiderska et al. [21] developed CNN models for automating transfusions of B lymph nodes based on HE-stained samples. They achieved an accuracy of 77% and an AUC of 83%. Sheng et al. [22] used an R-CNN network to identify lymphocytes from a malignant lymphoma dataset. It achieved a detection rate of 96% for malignant lymphocytes. Gaidano et al. [24] developed machine learning networks to diagnose DBCL from WSI images. They achieved an accuracy of 92.68% and sensitivity of 88.54%. Each study focuses on different aspects of lymphoma diagnosis and employs various methods and techniques. The performance metrics reported vary across the studies, including accuracy, sensitivity, AUC, and F1 score.

Comparing the performance of the proposed systems with the performance of the previous systems, it is seen that the performance of the proposed techniques is superior to the previous methods in all measures.

## 6. Conclusions

Manual diagnosis still faces many obstacles due to the similarity of biological characteristics among malignant lymphoma cells, especially in the early stages. Artificial intelligence techniques help doctors to distinguish between malignant lymphoma types. Several techniques have been developed between machine learning algorithms and hybrid deep learning networks to diagnose types of malignant lymphomas early. The images were enhanced by Gaussian and Laplacian filters to show the edges of the malignant lymphoma cells. The GVF algorithm was applied to extract the cells to be analyzed and isolate them from the rest of the image. The malignant lymphoma cells were then fed into three methodologies for diagnosing WSI images of malignant lymphoma. The first methodology for diagnosing malignant lymphoma cells is based on a hybrid model between the MobileNet, VGG16, and AlexNet networks to extract features and classify them using the XGBoost and DT algorithms. The second methodology for diagnosing malignant lymphoma cells using the XGBoost and DT algorithms is based on the combined features of the DL networks (MobileNet-VGG16, VGG16-AlexNet, and MobileNet-AlexNet). The third methodology for diagnosing malignant lymphoma cells is based on integrating the features of two models of DL networks with the handcrafted features and classifying them using the XGBoost and DT algorithms. The XGBoost network with fused features of MobileNet-VGG16 and handcrafted features has achieved an AUC of 99.43%, accuracy of 99.8%, precision of 99.77%, sensitivity of 99.7%, and specificity of 99.8%.

## Figures and Tables

**Table 1:** The strengths and weaknesses of the proposed system and its comparison with previous studies.

Approach	Strengths	Weaknesses
Irshad et al. [11]	Uses morphological parameters and features extracted from images to improve the accuracy of CNNs.	Requires good methods to extract features.
Xia et al. [12]	Uses a novel CNN architecture to improve the accuracy of lymphoma image classification.	Requires a large dataset of lymphoma images to train the CNN.
Savas et al. [13]	Uses a CNN-LSTM hybrid model to improve the accuracy of lymphoma image classification.	Requires a large dataset of lymphoma images to train the CNN-LSTM model.
Miyoshi et al. [14]	Uses deep learning networks to achieve high accuracy in the classification of lymphoma images with different magnification factors.	Requires a large dataset of lymphoma images with different magnification factors to train the deep learning networks.
Li et al. [15]	Uses a novel GOTDP-MP-CNNs network architecture to improve the accuracy of lymphoma image classification.	Requires a large dataset of lymphoma images to train the GOTDP-MP-CNNs network.
Syrykh et al. [16]	Uses Bayesian neural networks (BNNs) to improve the accuracy of lymphoma image classification.	Requires a large dataset of lymphoma images to train the BNNs.
Zhang et al. [17]	Uses a backpropagation (BP) network and genetic algorithm (GA) to improve the accuracy of lymphoma image classification.	Requires a large dataset of lymphoma images to train the BP-GA network.
Reena et al. [18]	Uses a feature-based image retrieval method to improve the accuracy of lymphoma image classification.	Requires a large dataset of lymphoma images to train the feature-based image retrieval method.
Zahra et al. [19]	Uses a novel method to discover the best deep learning methods for diagnosing lymphoma images.	Requires a large dataset of lymphoma images to train the deep learning methods.
Zhang et al. [20]	Uses a CNN model to achieve high accuracy in the classification of lymphoma images.	Requires a large dataset of lymphoma images to train the CNN model.
Swiderska et al. [21]	Uses a CNN model to automate the transfusion of B lymph nodes.	Requires a large dataset of B lymph node images to train the CNN model.
Sheng et al. [22]	Uses an R-CNN network to identify lymphocytes from lymphoma images.	Requires a large dataset of lymphoma images to train the R-CNN network.
Mohlman et al. [23]	Uses a CNN to distinguish between Burkitt lymphoma and diffuse large B-cell lymphoma (DLBCL).	Requires a large dataset of Burkitt lymphoma and DLBCL images to train the CNN.
Gaidano et al. [24]	Develops machine learning networks to diagnose DLBCL images collected from several sources.	Requires a large dataset of DLBCL images collected from several sources to train the machine learning networks.
Francisco et al. [25]	Develops a framework for extracting lymphoma biomarkers to determine their value compared to the rest of the nucleus.	Requires a large dataset of lymphoma images to train the framework.
Proposed method	Fuses deep learning models with color, texture, and shape features Extracts radiomics features from traditional methods.	Does not require expert input from doctors or a large dataset of lymphoma images to train the hybrid technique.

**Table 2:** Performance results of pre-trained DL models on diagnosing malignant lymphomas.

Models	Classes	AUC %	Accuracy %	Precision %	Sensitivity %	Specificity %
MobileNet	Lymph CLL	95.1	94.2	93.2	94.2	97.2
	Lymph FL	93.4	92.3	93.2	92.3	96.8
	Lymph MCL	92.4	91.7	91.8	91.9	95.8
	**Average ratio**	**93.63**	**92.70**	**92.73**	**92.80**	**96.60**
VGG16	Lymph CLL	94.3	93	93.7	93.2	96.7
	Lymph FL	91.5	93.3	91.7	92.8	96.5
	Lymph MCL	92.6	90.7	91.7	91.2	96.2
	**Average ratio**	**92.80**	**92.30**	**92.37**	**92.40**	**96.47**
AlexNet	Lymph CLL	92.9	92.8	94	93.2	96.8
	Lymph FL	93.1	92.3	91.4	91.7	96.2
	Lymph MCL	91.5	90.3	90.2	90.6	94.8
	**Average ratio**	**92.50**	**91.90**	**91.87**	**91.83**	**95.93**

**Table 3:** Performance results of hybrid models between DL and XGBoost for diagnosing malignant lymphomas.

Models	Classes	AUC %	Accuracy %	Precision %	Sensitivity %	Specificity %
MobileNet-XGBoost	Lymph CLL	96.8	97.2	95.7	97.2	97.8
	Lymph FL	97.2	95.6	97.5	96.1	98.7
	Lymph MCL	98.2	97.2	96.9	97.3	98.2
	**Average ratio**	**97.40**	**96.70**	**96.70**	**96.87**	**98.23**
VGG16-XGBoost	Lymph CLL	95.9	97.3	96.7	96.9	98.2
	Lymph FL	96.1	96	96.1	96.2	97.7
	Lymph MCL	96.7	95.8	96.3	95.8	97.6
	**Average ratio**	**96.23**	**96.40**	**96.37**	**96.30**	**97.83**
AlexNet-XGBoost	Lymph CLL	97.3	96	96	95.6	98.2
	Lymph FL	96.4	95.8	95.1	96.2	97.8
	Lymph MCL	96.1	95.7	96.4	95.8	97.6
	**Average ratio**	**96.60**	**95.80**	**95.83**	**95.87**	**97.87**

**Table 4:** Performance results of hybrid models between DL and DT for diagnosing malignant lymphomas.

Models	Classes	AUC %	Accuracy %	Precision %	Sensitivity %	Specificity %
MobileNet-DT	Lymph CLL	95.1	95.3	94.4	95.1	97.1
	Lymph FL	95.9	94.7	94.2	95.4	96.8
	Lymph MCL	96.5	94.3	95.6	94.3	98.2
	**Average ratio**	**95.83**	**94.80**	**94.73**	**94.93**	**97.37**
VGG16-DT	Lymph CLL	96.8	95.2	93.8	95.2	96.8
	Lymph FL	94.7	93.24	93.9	93.1	97.1
	Lymph MCL	95.8	94.6	95.6	94.8	97.6
	**Average ratio**	**95.77**	**94.40**	**94.43**	**94.37**	**97.17**
AlexNet-DT	Lymph CLL	96.1	94.7	93.6	94.8	97.4
	Lymph FL	95.8	93.4	93.1	93.2	96.8
	Lymph MCL	94.7	92.8	94.2	92.7	97.1
	**Average ratio**	**95.53**	**93.80**	**93.63**	**93.57**	**97.10**

**Table 5:** Performance results of XGBoost with features fused to DL for the diagnosis of malignant lymphomas.

Models	Classes	AUC %	Accuracy %	Precision %	Sensitivity %	Specificity %
MobileNet-VGG16-XGBoost	Lymph CLL	98.7	99	98.2	98.8	99.1
	Lymph FL	97.9	97.7	98.8	97.9	98.7
	Lymph MCL	98.2	98.8	98.5	98.5	98.6
	**Average ratio**	**98.27**	**98.50**	**98.50**	**98.40**	**98.80**
VGG16-AlexNet-XGBoost	Lymph CLL	98.6	98.9	98.7	99.1	98.6
	Lymph FL	98.5	98.2	98.1	98.2	99.2
	Lymph MCL	97.8	98	98.3	97.9	98.8
	**Average ratio**	**98.30**	**98.40**	**98.37**	**98.40**	**98.87**
MobileNet-AlexNet-XGBoost	Lymph CLL	98.7	99	98.4	98.7	98.9
	Lymph FL	97.6	98.1	97.8	97.9	99.2
	Lymph MCL	97.1	97.6	98.5	98.2	98.7
	**Average ratio**	**97.80**	**98.20**	**98.23**	**98.27**	**98.93**

**Table 6:** Performance results of the DT algorithm with features fused to DL for the diagnosis of malignant lymphomas.

Models	Classes	AUC %	Accuracy %	Precision %	Sensitivity %	Specificity %
MobileNet-VGG16-DT	Lymph CLL	98.1	98.2	97.6	98.2	98.7
	Lymph FL	97.9	97.1	97.5	97.1	99.1
	Lymph MCL	98.3	98.1	98.3	98.3	98.6
	**Average ratio**	**98.10**	**97.80**	**97.80**	**97.87**	**98.80**
VGG16-AlexNet-DT	Lymph CLL	98.3	97.8	97.7	98.2	98.9
	Lymph FL	98.6	97.8	97.3	98.1	99.3
	Lymph MCL	97.5	97.4	98	96.8	98.7
	**Average ratio**	**98.13**	**97.70**	**97.67**	**97.70**	**98.97**
MobileNet-AlexNet-DT	Lymph CLL	98.8	97.5	97.5	97.2	98.5
	Lymph FL	98.3	97.3	97.6	96.8	99.1
	Lymph MCL	98.1	97.2	96.9	96.5	97.8
	**Average ratio**	**98.40**	**97.30**	**97.33**	**96.83**	**98.47**

**Table 7:** Performance results of XGBoost with features fused to DL and handcrafted for the diagnosis of malignant lymphomas.

Classifier	Fusion Features	Classes	AUC %	Accuracy %	Precision %	Sensitivity %	Specificity %
XGBoost algorithm	MobileNet-VGG16-Handcrafted	Lymph CLL	99.2	99.8	99.5	99.7	99.9
		Lymph FL	99.5	99.8	100	99.8	99.8
		Lymph MCL	99.6	99.7	99.8	99.6	99.7
		**Average ratio**	**99.43**	**99.80**	**99.77**	**99.70**	**99.80**
	VGG16-AlexNet-Handcrafted	Lymph CLL	99.4	99.6	99.6	99.5	99.8
		Lymph FL	99.6	100	99.8	99.7	99.6
		Lymph MCL	99.7	99.6	99.8	99.8	99.7
		**Average ratio**	**99.57**	**99.70**	**99.73**	**99.67**	**99.70**
	MobileNet-AlexNet-Handcrafted	Lymph CLL	99.6	99.7	99.5	99.5	99.8
		Lymph FL	99.1	99.2	99.8	99.1	99.7
		Lymph MCL	99.5	99.6	99.2	99.7	99.6
		**Average ratio**	**99.40**	**99.50**	**99.50**	**99.43**	**99.70**

**Table 8:** Performance results of DT algorithm with features fused to DL and handcrafted for the diagnosis of malignant lymphomas.

Classifier	Fusion Features	Classes	AUC %	Accuracy %	Precision %	Sensitivity %	Specificity %
DT algorithm	MobileNet-VGG16-Handcrafted	Lymph CLL	99.5	99.3	99.2	99.1	99.5
		Lymph FL	99.4	99.3	99.5	99.2	99.8
		Lymph MCL	99.2	99.2	99.1	98.8	99.7
		**Average ratio**	**99.37**	**99.30**	**99.27**	**99.03**	**99.67**
	VGG16-AlexNet-Handcrafted	Lymph CLL	98.9	98.8	99.3	99.2	99.8
		Lymph FL	98.5	98.8	98.4	99.1	99.1
		Lymph MCL	98.2	98.6	98.5	98.7	98.7
		**Average ratio**	**98.53**	**98.70**	**98.73**	**99.00**	**99.20**
	MobileNet-AlexNet-Handcrafted	Lymph CLL	99.2	98.9	99	99.2	98.8
		Lymph FL	98.9	98.7	98.6	98.9	99.2
		Lymph MCL	99	98.7	98.7	99.1	99.4
		**Average ratio**	**99.03**	**98.80**	**98.77**	**99.07**	**99.13**

## Data Availability

In this study, data supporting the performance of the proposed systems were collected from a dataset that is accessible to the public, researchers, and those interested at the link: https://www.kaggle.com/datasets/obulisainaren/multi-cancer (accessed on 15 October 2022).
